# A Single Dose of Oral Sucrose Is Enough to Control Pain During Venipuncture: A Randomized Controlled Trial

**DOI:** 10.3389/fpain.2022.888076

**Published:** 2022-05-11

**Authors:** Maria Elena Cavicchiolo, Marco Daverio, Nadia Battajon, Anna Chiara Frigo, Paola Lago

**Affiliations:** ^1^Neonatal Intensive Care Unit, Department of Woman's and Child's Health, University of Padua, Padua, Italy; ^2^Paediatric Intensive Care Unit, Department of Woman's and Child's Health, University of Padua, Padua, Italy; ^3^Neonatal Intensive Care Unit, Azienda Unità Locale Socio Sanitaria (ULSS) 2 Marca Trevigiana, Treviso, Italy; ^4^Department of Cardiac-Thoracic-Vascular Sciences and Public Health, University of Padua, Padua, Italy

**Keywords:** preterm, pain, skin breaking procedures, sucrose, non-pharmacologic treatment

## Abstract

Sucrose is effective in reducing pain during minor procedures in neonates. We evaluated whether a second dose of sucrose was more effective than a single dose during venipuncture. We performed a randomised, double-blind, controlled trial at the NICU of Padua Hospital (August 2016-October 2017). We randomised 72 preterm infants undergoing venipuncture for routine test to a control group, which received a single standard dose of sucrose 2′ before the procedure and a placebo 30″ after the venipuncture, and an experimental group in which they received two doses of 24% sucrose 2′ before and 30″ after the venipuncture. No difference in pain perception was found between the groups at 30″, 60″ and 120″. In conclusion, we do not recommend a second dose of sucrose during venipuncture in prematures.

## Short Communication

Skin breaking procedures in neonatology are defined as all the procedures producing a skin lesion, including heel prick, venipuncture and vaccine administration (1). Although these procedures vary in terms of duration and intensity of pain, the guidelines tend to group them under the abovementioned “skin breaking” definition and advise the use of non-pharmacological treatments, such as the use of sucrose, for the prevention of all procedural pain in neonates (2, 3). It has been demonstrated that oral sucrose, in association with non-nutritive sucking and facilitated tucking, is effective in reducing pain during minor procedures in neonates and preterm babies and it is now included in almost all international position papers and recommendations for the treatment of mild to moderate procedural pain (4–8).

The Neosucrose study conducted by our group, demonstrated that a second dose of 24% sucrose was not superior to a single dose in reducing pain during the recovery phase of heel prick (9), but no studies compared these two pain control strategies for venipuncture.

Previous studies reported that venipuncture was a less painful and more effective skin breaking procedure than heel prick for blood sample procurement in expert hands, but these results dated to more than 15 years ago and included patients receiving a heel prick with manual lancets (10, 11). No data are available comparing venipunctures and heel pricks performed with automatic lancets, which are capable of different cutting width for infants according to their weight. Therefore, we primarily aimed to evaluate whether a second dose of sucrose (e.g., 0.3–0.5 mL) was more effective than a standard single dose in pain control during venipuncture, as already done for heel prick. As a secondary aim, we compared the pain response in patients receiving a venipuncture or a heel prick undergoing the same experimental conditions, already published.

We performed a randomized, double-blind, controlled trial (RCT) at the Neonatal Intensive Care Unit (NICU) of the Department of Woman's and Child's Health, University Hospital of Padua, between August 2016 and October 2017. All preterm infants (<37 weeks gestational age) admitted to the NICU with a postnatal age <28 days or before reaching 40 weeks corrected postmenstrual age undergoing a venipuncture for a routine test, were eligible for enrolment. We randomized patient to a control group, which received a single standard dose of sucrose (24%, 0.3 mL for a total of 0.07 g of sucrose for infant ≤ 1,000 g or 0.5 mL, 0.12 g for infants >1,000 g) 2 min before the procedure and a placebo (distilled water) 30 seconds after the venipuncture, and an experimental group in which the preterm infants received two doses of 24% sucrose 2 min before and 30 s after the venipuncture. Sucrose and placebo syringes were shielded by the research nurse in order to blind the bedside nurse and video recorded (digital video).

All venipunctures were performed by the patient's nurse according to a standardized unit check-list made up of three phases which were all videotaped: (1) Baseline phase, (2) Venipuncture phase, (3) Recovery phase. Data collection started during the baseline phase, before any manipulation. Sucrose was given directly on the tongue with the syringe, 2 min before the procedure together with a pacifier as per NICU standard practice. After accurate skin preparation, venipuncture was performed using a winged infusion set. We used a 24-gauge butterfly needle for all procedures and the sites for blood collection were the cubital fossa of the forearm and head superficial veins. In the third phase, lasting 2 min after the procedure, the neonate was monitored until the vital signs returned to the baseline status.

As secondary analysis we considered the entire population of the NeosucroseTrial including 139 patients, 71 receiving a heel prick (see reference 8 for complete methods description) and 68 a venipuncture as skin breaking procedure and compared the pain response of the infants undergoing heel prick procedures to those who experienced venipuncture. For both analysis, pain level was evaluated using the Premature Infant Pain Profile (PIPP) scale (12). A PIPP score <7 was considered no Pain, PIPP score between 7.0 and 12.0 mild to moderate pain and a score >12.0 severe pain.

The patient's demographic and clinical characteristics have been described by using mean and standard deviation or median, minimum and maximum for whether the data distribution was considered.

The patient's demographic and pain data were compared between treatment groups with Student's test or Wilcoxon's ranks sum test, depending on whether the distribution of quantitative variables was considered normal or not (QQ plot and Shapiro-Wilk's test) and with Chi-square or Fisher's exact test, when appropriate, for qualitative variables. All data were analyzed using the SAS 9.4 (SAS Institute Inc., Cary, NC, USA) for Windows.

Written informed consent was obtained from the infant's caregiver prior to enrolment and the study was approved by the Hospital Ethics Committee (reference number 3714/AO/2016) and registered in clinicaltrials.gov (NCT 02859376).

In the study period, we randomized 72 infants in the venipuncture group for a final sample of 68 preterm infants. Four patients were excluded for poor quality of videorecording, for a final population of 68 videos. There were no statistical differences between the group receiving a second dose of 24% sucrose and the group receiving a single dose, in relation to patient characteristics at enrolment. Venipunctures were performed over the scalp in fifty-two patients (76%) and in the arms for 16 preterms (24%) with same distribution in both groups. No second attempts were needed for blood collection.

No difference in pain response was found between the groups at 30, 60 and 120 s: at 30 s the median PIPP was 6.5 (interquartile range [IQR] 1.0–15.0) in the experimental group vs. 6.0 (IQR 1.0–13.0) in the control group; at 60 s the median PIPP was 5.5 (IQR 0.0–15.0) in the experimental group vs. 5.0 (IQR 1.0–10.0) in the control group; at 120 s, the median PIPP in the experimental group was 5.0 (IQR 0.0–15.0) vs. 6.0 (IQR 0.0–17.0) in the control group (Table 1).

**Table 1:** Patient characteristic and PIPP scores at 30 s, 60 s,120 s in both groups of treatment.

	**Experimental group**	**Control** **group**	***p-value***
	**(*N* = 34)**	**(*N* = 34)**	
Gestational weeks at birth, mean (SD)	29.8 (2.8)	28.1 (2.9)	0.0173^°^
Birth weight (grams), median (min-max)	1195.0 (515.0–2375.0)	1142.5 (630.0–2435.0)	0.288^a^
Apgar score 5th minute, median (min-max)	8.0 (4.0–9.0)	8.0 (4.0–9.0)	0.408^a^
Sex Male *n* (%)	14 (41.2)	16 (47)	0.807^b^
Cesarean delivery *n* (%)	25 (73.5)	29 (85.3)	0.260^c^
Antenatal steroids *n* (%)	28 (82.4)	28 (82.4)	1.000^c^
Gestational weeks at the procedure, mean ± SD	33.1 ± 2.9	31.9 ± 3.6	0.115°
Days of life at the procedure, Median (min-max)	17.0 (1.0–80.0)	20.5 (1.0–81.0)	0.585^a^
PIPP score at 30 s, median (IQR)	6.5 (1.0–15.0)	6.0 (1.0–13.0)	0.639^a^
PIPP score at 60 s, median (IQR)	5.5 (0.0–15.0)	5.0 (1.0–10.0)	0.790^a^
PIPP score at 120 s, median (IQR)	5.0 (0.0–15.0)	6.0 (0.0–17.0)	0.158^a^

° *Student's t-test,*

a *Wilcoxon rank-sum test,*

b *Chi-square test,*

c *Fisher's exact test*.

Comparing the pain response of patients receiving a heel prick or a venipuncture in the same experimental conditions, heel prick was significantly more painful than venipuncture at 60 s (median PIPP 6.0 [IQR 1-14] vs. 5.0 [IQR 0-15], *p* = 0.01) but this was not shown at the other time points (Table 2).

**Table 2:** Frequency of pain intensity by severity.

	**Heel prick group**	**Venipuncture group**	***p*-value**
	***N* = 71**	***N* = 68**	
PIPP^a^ score at 30 s, median (range)	7.0 (2.0–16.0)	6.0 (1.0–15.0)	0.144^b^
PIPP^a^ score at 60 s, median (range)	6.0 (1.0–14.0)	5.0 (0.0–15.0)	**0.01^b^**
PIPP^a^ score at 120 s, median (range)	6.0 (1.0–17.0)	5.0 (0.0–17.0)	0.08^b^

a *PIPP: Premature Infant Pain Profile.*

b *Wilcoxon rank-sum test.*

*The bold values indicate the values which is statistically significant (p < 0.05)*.

Our study demonstrates that a second dose of sucrose significantly lower the pain scores of preterm infants receiving a venipuncture. A single dose a single dose of 24% sucrose with non-nutritive sucking administered before the procedure is as effective at reducing pain scores in response to both skin breaking procedures compared to two doses of 24% sucrose (9).

As for our secondary outcome in our sample of preterm infants, the heel prick procedure was significantly more painful than the venipuncture at 60 s. It is possible that this difference at this timepoint can be due to the heel squeeze performed after a heel stick procedure to obtain blood. The clinical relevance of this finding is debatable, since the median PIPP of heel prick was one point higher than venipuncture and a significant difference in pain score is defined as a reduction of PIPP score of at least two points. Therefore, it is still not clear whether heel prick is more painful than venipuncture in infants exposed to the same experimental conditions and further studies are needed to better clarify this aspect.

We recognize some study limitations. First of all a higher number of patients would be required to have more conclusive data, second, the length of the procedure: venipuncture is an operator-dependent procedure, the duration and the intensity may be different depending on the nurse who performed the procedure. Finally, we did not explore the effect of sucrose at other time points (e. g., 60 s).

In conclusion, we do not recommend a second dose of sucrose during skin breaking procedures in preterm infants admitted to NICU.

## Ethics Statement

This study was reviewed and approved by the Hospital Ethics Committee (reference number 3714/AO/2016) and registered in clinicaltrials.gov (NCT 02859376). Written informed consent was obtained from the infant's caregiver prior to enrolment.

## Author Contributions

PL conceived the study and wrote the first draft of the manuscript. MC wrote the final version of the manuscript. MC and NB evaluated the videos in collaboration with PL. MD elaborated the statistical analysis with the supervision of AF, who wrote the methodological part of the manuscript. All authors approved the final version of the manuscript.

## Conflict of Interest

The authors declare that the research was conducted in the absence of any commercial or financial relationships that could be construed as a potential conflict of interest.

## Publisher's Note

All claims expressed in this article are solely those of the authors and do not necessarily represent those of their affiliated organizations, or those of the publisher, the editors and the reviewers. Any product that may be evaluated in this article, or claim that may be made by its manufacturer, is not guaranteed or endorsed by the publisher.
